# Sigmoid diverticulitis: US findings

**DOI:** 10.1186/2036-7902-5-S1-S5

**Published:** 2013-07-15

**Authors:** Maria Antonietta Mazzei, Nevada Cioffi Squitieri, Susanna Guerrini, Amato Antonio Stabile Ianora, Lucio Cagini, Luca Macarini, Melchiore Giganti, Luca Volterrani

**Affiliations:** 1 Department of Medical, Surgical and Neuro Sciences, Section of Radiological Sciences. Siena, Italy; 2 University of Bari, Diagnostic Imaging Section, Bari, Italy; 3 University of Perugia, Thoracic Surgery Unit, Perugia, Italy; 4 University of Foggia, Department of Radiology, Foggia, Italy; 5 University of Ferrara, Dipartimento di Scienze Chirurgiche, Ferrara, Italy

## Abstract

Acute diverticulitis (AD) results from inflammation of a colonic diverticulum. It is the most common cause of acute left lower-quadrant pain in adults and represents a common reason for acute hospitalization, as it affects over half of the population over 65 years with a prevalence that increases with age. Although 85% of colonic diverticulitis will recover with a nonoperative treatment, some patients may have complications such as abscesses, fistulas, obstruction, and /or perforation at presentation. For these reasons, different classifications were introduced through times to help clinicians to develop a correct diagnosis and guide the treatment and for the same reasons imaging is used in most cases both to realise a differential diagnosis and to guide the therapeutic management. US and CT are both usefull in diagnosis of diverticolitis, and their sensibility and specificity are similar. However CT scanning is essential for investigating complicated diverticular disease especially where there are diffuse signs and clinical suspicion of secondary peritonitis; instead in most uncomplicated cases the experienced sonographer may quickly confirm a diagnosis guided by the clinical signs. US is to be recommended in premenopausal women, and in young people to reduce dose exposure.

## Background

Acute diverticulitis (AD) results from inflammation of a colonic diverticulum. It is the most common cause of acute left lower-quadrant pain in adults and represents a common reason for acute hospitalization, as it affects over half of the population over 65 years with a prevalence that increases with age [1,2]. Although 85% of colonic diverticulitis will recover with a non operative treatment, some patients may have complications such as abscesses, fistulas, obstruction, and /or perforation at presentation [3] . Moreover clinical differentiation from other causes of abdominopelvic pain is often difficult (Table 1), so imaging is used in most cases both to realise a differential diagnosis and to guide the therapeutic management [4]. Hollerweger A et al realised an alternative diagnosis in 47 out of 175 patients clinically suspected of having diverticulitis, with a relatively high frequency of epiploic appendagitis (8 out of 47 patients), ureterolitihasis (6 out of 47 patients), urinary tract infection and pelvic inflammatory disease (4 out of 47 patients), and other bowel pathologies, as ischemic colitis, infectious enterocolitis and perforated carcinoma (3 out of 47 patients) [5]. Currently, Multidetector Computed tomography (MDCT) of the abdomen is often the diagnostic test of choice, especially in the urgent assessment of patients with AD, with sensitivity, specificity, and positive and negative predictive values all well greater than 95%, allowing the identification of both colonic abnormalities (particularly mural thickening and diverticula) and inflammatory changes in the pericolonic fat planes, grading its severity, and identifying most usual complications [6-8]. The usual MDCT features include segmental wall thickening with spasm, submucosal oedema, diverticula, vascular engorgement, and inflammatory changes in the pericolic fat, plus fascial, mesenteric, and/or peritoneal fluid [6]. Conventional contrast-enhanced MDCT is also highly reliable in excluding an underlying carcinoma [9,10]. Some Authors claims that CT sensitivity is higher than that of ultrasound (US) in detecting diverticulitis (81% versus 61%, p = 0.048) in unselected patients presenting with acute abdominal pain, but the positive predictive values are comparable, whereas in presence of clinical suspicion of diverticulitis the sensitivity of US reaches the 92% [11-15]. In particular US is highly sensitive and specific for uncomplicated acute diverticulitis and for the primary complication of pericolic abscess [16-18]. In this review, we discuss the US findings of diverticulitis and the role of US in it’s diagnostic management.

**Table 1:** Possible alternative diagnosis of Left Lower-Quadrant Pain.

Gastrointestinal	Genitourinary/gynecologic	Vascular/Other
Epiploic appendagitis	Ureterolithiasis	Dissection/ruptured aneurysm
Ischemic colitis	Urinary tract infection	Abdominal wall abscess
Infectious enterocolitis	Ectopic pregnancy	Abdominal wall hematoma
Perforated carcinoma	Hemorrhagic or rupture ovarian cyst	Psoas abscess
Small bowel obstruction	Ovarian torsion	Retroperitoneal hemorrhage
Inflammatory bowel disease	Ruptured corpus luteum	
Appendicitis	Uterine fibroids torsion	
Small bowel infarction	Pelvic Inflammatory disease	
Omental infarction		
Incarcerated hernia		

## Main body

### Pathogenesis and classifications

Diverticulitis results from occlusion of a colonic diverticulum by stool, inflammation, or food particles, causing a microperforation and surrounding pericolic inflammation. A colonic diverticulum is a herniation of mucosa and submucosa, corresponding to a weak point where the vasa recti penetrate the tunica muscularis, so most colonic diverticula are “false diverticula” containing no muscularis propria. In 1965 Painter et al. presented the hypothesis that diverticular disease was caused by excess pressure in the colon due to segmentation based on insufficient intake of dietary fibre [19]. In the Western world diverticulosis occurs primarily in the sigmoid colon, corresponding to the highest intraluminal pressure, where they are often associated with other typical findings of diverticular disease (i.e. muscularis propria thickening, shortening and narrowing of the lumen). The incidence for diverticulosis is 33–66%, of these patients, 10–25% will develop an acute episode of diverticulitis [2]. Other risk factors are: obesity (BMI≥30), use of non-steroidal anti-inflammatory drugs or acetaminophen; smoking was not significantly associated with symptomatic diverticular disease, and a genetic predisposition was not proved yet [20]. Diverticula vary in size from tiny intramural and transient phenomena to permanent protrusions up to several centimeters in diameter. Retention of fecal matter within the diverticulum due to an occlusion may produce a mucosal abrasion resulting in infection or inflammation of the diverticulum wall (diverticulitis). The process may realise a focal intramural inflammatory mass or abscess, infiltrate along the bowel wall to produce an inflammatory bowel segment, and perforate into sigmoid mesentery where the process is usually contained. Therefore its presentation may vary greatly per individual patient, from symptomatic diverticulosis to perforated diverticulitis [21-23], even if the majority of individuals with diverticulosis are asymptomatic [24-26]. However, perforation can cause intraperitoneal contamination that is associated with a much higher morbidity and mortality. From 1978 until today different classifications were proposed for the staging of acute complicated diverticolitis; first Hinchey that proposed a classification in 4 stages, developed to predict outcomes following the surgical management of complicated diverticular disease (perforated disease) (stage I: mesocolic/pericolic abscess; stage II: pelvic abscess; stage III generalized peritonitis; stage IV faecal peritonitis) [27]. Then the much more detailed information provided by CT scans led earlier to modifications of the original Hinchey classification. Subcategories could be defined considering the radiological findings. In 1997 Sher et al introduced the first modification for distinguishing between pericolic abscesses (stage I), distant abscesses amendable for percutaneous drainage (stage IIa), and complex abscesses associated with a possible fistula (stage IIb). This modification also implied the use of new treatment strategies such as CT-guided percutaneous drainage of abscesses (Table 2) [28-30] . From these, several other classifications have been made (Wasvary et al. in 1999, Kohler et al in 1999, Siewert et al in 1995), among that deserve mention the Hansen/Stock classification and Kohler et al classification, both based on the clinical severity and presentation of disease (Table 3) [31,32]. Finally in 2002 Ambrosetti et al. proposed a simplified staging of acute diverticulitis based on CT criteria and showed its prognostic significance in a prospective study: moderate diverticulitis defined by localized sigmoid wall thickening (<5mm) with pericolic fat stranding, and severe diverticulitis defined by wall thickening accompanied by abscess, extraluminal air or extraluminal contrast [33]. More recently Klarenbeek et al have proposed a new classification arranged in three stages of differentiating diverticula disease: a) uncomplicated, b) chronic complicated, and c) acute complicated, and according both clinical and radiological findings in addition to treatment modalities, in order to guide the clinical management and form the basis of a practice parameter for diverticular disease [16].

**Table 2:** Hinchey classification and modified Hinchey classification by Sher et al.

Hinchey classification	Modified Hinchey classification by Sher et al
I pericolic abscess or phlegmon	I pericolic abscess
II pelvic, intrabdominal or retroperitoneal abscess	IIa distant abscess amendable to percutaneous drainage
	IIb Complex absess associated with fistula
III Generalized purulent peritonitis	III Generalized purulent peritonitis
IV Generalized fecal peritonitis	IV Fecal peritonitis

**Table 3:** Köhler and Hansen/Stock classification.

Köhler Classification	Hansen/Stock classification
Symptomatic uncomplicated disease	0 Diverticulosis
Recurrent symptomatic disease	I Acute uncomplicated diverticulitis
Complicated disease:	II Acute complicated diverticulitis
Hemorrhage, Abscess, Phlegmon	a. Phlegmon, peridiverticulitis
Fistula, Perforation, Stricture	b. Abscess, sealed perforation
Purulent and fecal peritonitis, Small bowel	c. Free perforation
obstruction due to post-inflammatory adhesions	III Chronic recurrent diverticulitis

### Clinical presentation and treatment

From a clinical point of view, comparing patients with non specific abdominal pain to those with diverticulitis, they are more likely to have a subacute onset of pain (>1 hr), tenderness to palpation only in the left lower quadrant, and raised inflammatory markers; and are less likely to have nausea and vomiting [34]. Other symptoms are: fever, absence of peristalsis, and defence muscularis. Diverticular bleeding and pneumaturia (pathognomic for a colovesical fistula) are rare.

The wide spectrum of diverticular disease requires a differentiated therapeutic approach to the different manifestations. Moderate cases of diverticular disease (phlegmon or small abscess) can be treated conservatively, generally with antibiotics and an easily digestible diet. Large abscesses, if amendable and usually larger than 5 cm, should be good candidates for CT-guided percutaneous drainage [35]. This procedure may relieve symptoms or function as a bridge to (elective) surgery. In cases of fecal peritonitis resulting from a perforation acute surgical intervention should be warranted because it is associated with high morbidity and mortality (10–35%) [36]. It is now thought that after a conservatively treated episode, diverticular disease usually follows a rather benign course and that complications occur mostly at first presentation [37,38]. Therefore, elective sigmoid resections should be restricted for use in treating complicated disease, such as symptomatic stenosis, fistulas to a hollow organ, or recurrent diverticular bleeding [39].

### Imaging

**Figure 1:**
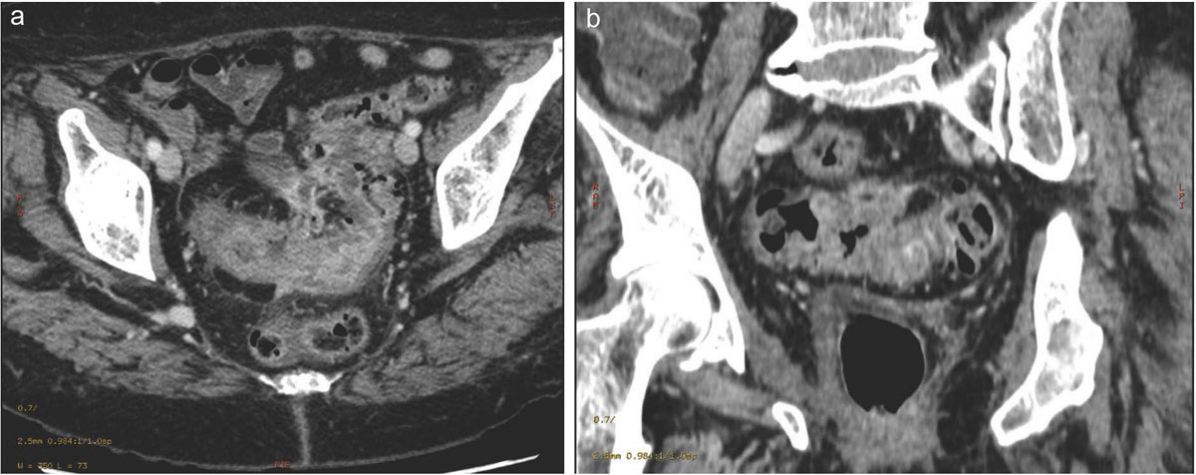
Contrast-enhanced MDCT 2D reconstruction on axial –oblique (a) and coronal oblique (b) planes shows a neoplastic thickening of the colonic wall (sigmoid tract) in presence of diverticulitisis.

Imaging may not be necessary in patients with the classic triad of left lower-quadrant pain, fever, and leukocytosis, and in whom uncomplicated diverticulitis is suspected. Imaging also may not be necessary in patients with a history of diverticulitis who present with relatively mild clinical symptoms of recurrent disease. However, considering the wide spectrum of diverticular disease , also depending on the age of patients (clinical presentation may differ significantly in older patients where a reduction in symptom severity causing a misdiagnosis could be present), and the possibile difficult differential diagnosis from other causes of abdominopelvic pain, imaging often plays a definitive role both to realise a differential diagnosis and to guide the therapeutic management [1,8,40]. As reported above, today contrast-enhanced CT has a pivotal role in the clinical practice regarding diverticular disease, and because of their superior sensitivity and specificity up to 100%, replaced the most important imaging modality [40]. Especially when an associated abscess is suspected, a CT scan can be very helpful to demonstrate its presence, and also for evaluate the possibility of a percutaneous drainage [41]. In the case of diverticular bleeding, a contrast-enhanced CT examination may demonstrate a contrast blush; and even if it has to be considered that 80% of all diverticular bleeding is self-limiting, occasionally successes of highly selective arterial embolization are described. Although the role of interventional radiology is yet to be determined. Conventional contrast-enhanced MDCT is also highly reliable in excluding other diagnoses of acute abdomen and an underlying carcinoma (Figure 1) [14,15,42-44]. A colonoscopy is indicated when there is doubt about cancer, persisting or recurrent complaints in the left lower quadrant, and suspicion of a stenosis or recurrent blood loss. Follow-up colonoscopy for ruling out malignancy is usually performed 6 weeks after an episode of acute diverticulitis. Moreover the computed tomography colonography (CTC) seems to be a reasonable alternative in follow-up of patients with symptomatic diverticular disease [45]. In recent years, magnetic resonance imaging (MRI) has gained popularity, because it lacks the ionizing radiation and even if CT remains the modality of choice; however MRI can similarly demonstrate findings of diverticulitis and could be useful in diagnosis of ischemic colitis [46,47]. Although, colonic diverticulitis is easily diagnosed and classified (graded) by CT than by ultrasound (US), it is important to be aware of the US signs of diverticulitis considering that US is often used as a first modality in the diagnostic approach to the acute abdomen.

### US: techinique and findings

US is an imaging modality widely available in the Emergency Department. The lower cost and in particular the lack of radiation exposure are the most important advantages of US compared to CT. Furthermore US is a real-time dynamic examination and this characteristic conveys dynamic information about bowel motility and changes in position, and to depict blood flow. Another important advantage of US examination is the possibility to correlate the US findings with the point of maximal tenderness. Besides, in not too obese patients, US may be superior to CT, and it is most useful in early, uncomplicated diverticulitis. From a technical point of view , both curved (3.5–5.0-MHz) and linear (5.0 –12.0-MHz) transducers are most commonly used, in particular the higher frequency probes are useful for detailed investigation of the large bowel wall, identifying its typical haustral pattern. Focal bowel masses, segments of wall thickening, and dilated loops may be evident even at lower frequencies, but high-frequency probes are essential to identify and chracterize changes in the layers of the bowel wall. At higher frequencies, US images the bowel wall as five alternating bands (gut signature) of high and low echogenicity to produce a characteristic sonographic signature approximating to the concentric layered histologic structure [48]. Visualization of the fine inner and outer bright layers (interface echoes) is highly dependent on the echogenicity of adjacent structures and is most easily seen where there is fluid in the bowel lumen or ascites between bowel loops. Even at lower diagnostic frequencies in loops further from the probe, at least the two most prominent layers are evident due to their relative thickness and high contrast: the bright submucosa (third layer) and the dark muscularis propria (fourth layer) [34]. Doppler scanning demonstrates no signal in normal bowel wall [49,50].

**Figure 2:**
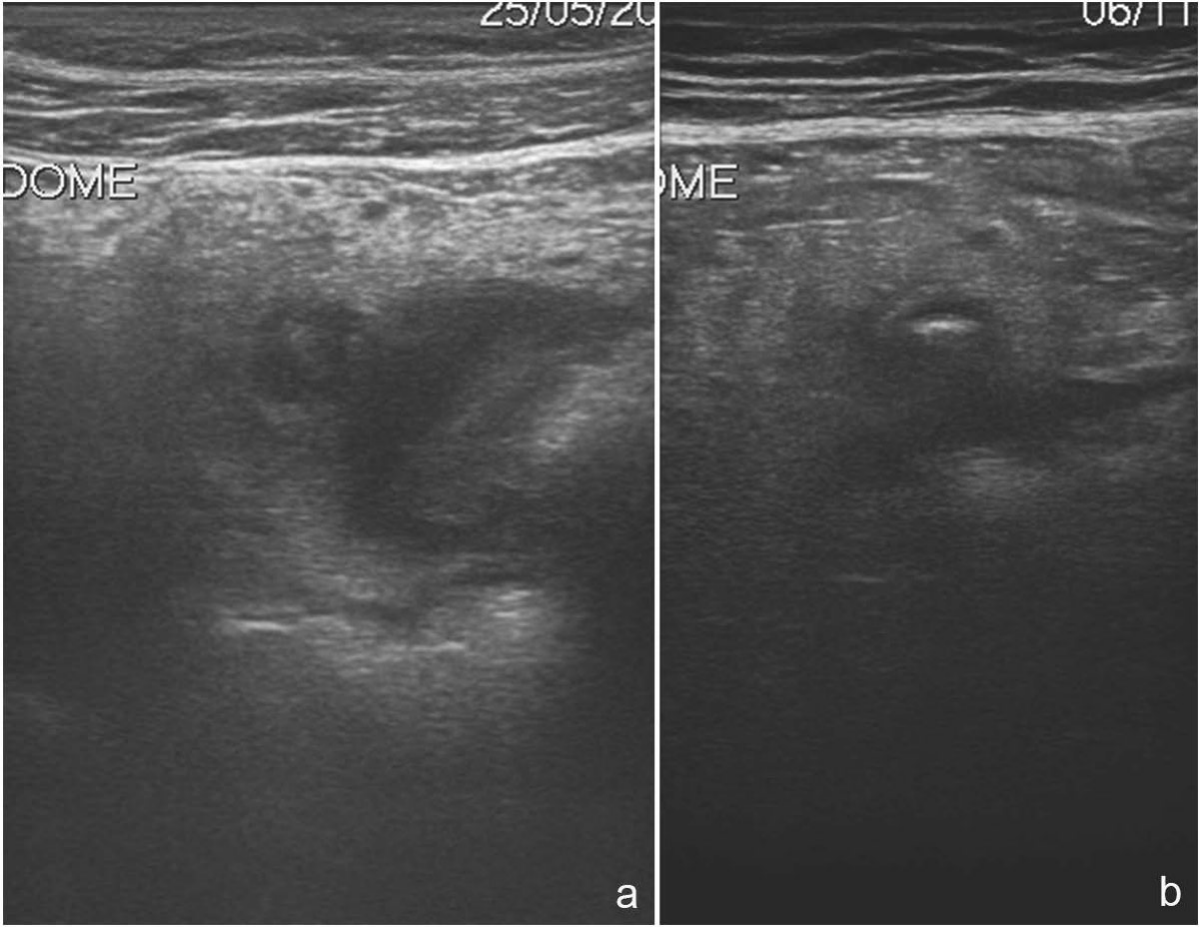
Sonographic features of uncomplicated diverticulitis: diverticula appear as bright “ear” out of the bowel wall (a); a central shadowing echogenicity may indicate the presence of fecalith (b).

**Figure 3:**
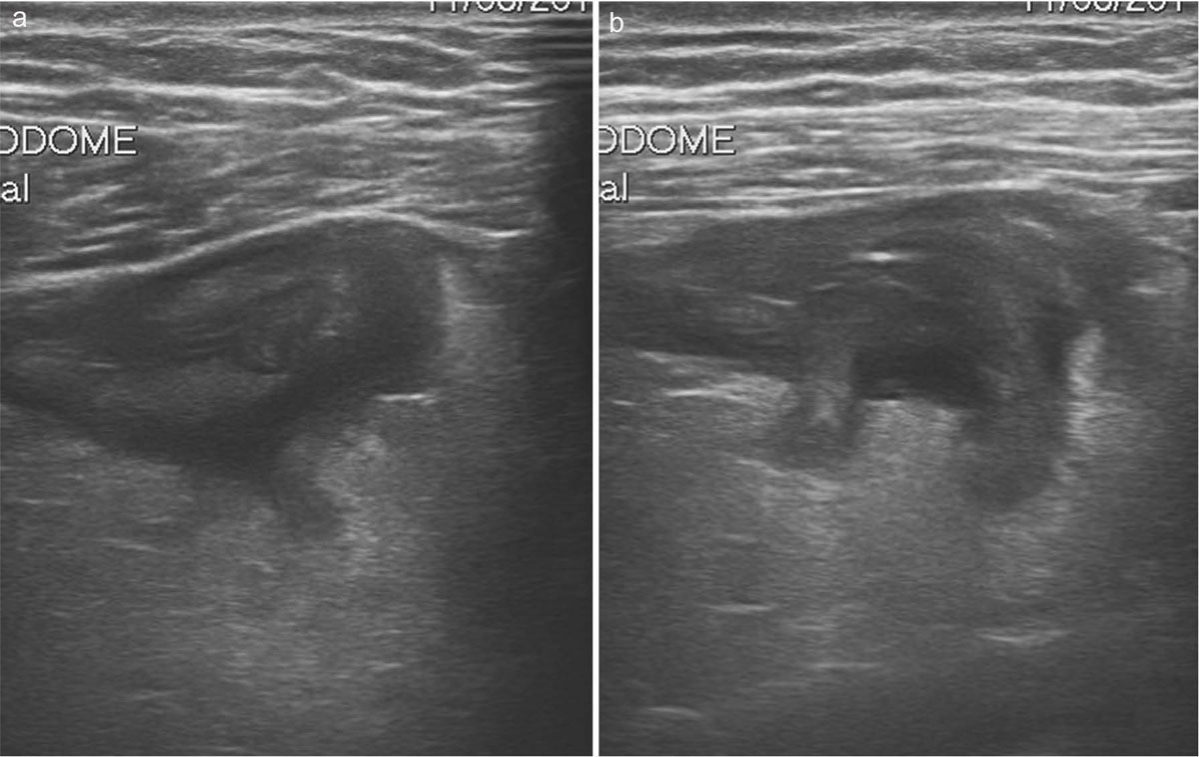
Sonographic features of complicated diverticulitis: the images show the presence of diveticula, thickening of the bowel wall and pericolic fluid (a,b)

A thinned diverticular wall may be demonstrated at higher probe frequencies with a reduced gut signature due to the absence of muscularis propria. Diverticula appear as bright “ears” out of the bowel wall with acoustic shadowing due to the presence of gas or inspissated feces; furthermore the neck of diverticulum may be identified as an echogenic band traversing hypoechoic muscularis propria that is often thickened. The diagnostic criteria for diverticulitis that has been used in US and CT are the same: 1) at least one diverticulum, 2) signs of inflammation of periodic fat (dirty fat/stranding) and 3) thickened bowel wall > 4-5 mm [33]. An isolated inflamed diverticulum is identified as an enlarged echo-poor protrusion from the colon wall, with an ill-defined margin surrounded by echogenic noncompressible fat, which represents the inflamed mesentery and omentum 'sealing off' the imminent perforation. The diverticulum wall signature is lost. A central shadowing echogenicity may indicate the presence of fecalith (Figure 2) [51]. Often the inflammation will have extended into the bowel producing asymmetrical or circumferential hypoechoic mural thickening that may demonstrate hyperemia on Doppler scanning. Diverticulitis may progress to an intramural or pericolic abscess indicated by an anechoic collection that may contain pockets of air or debris (Figure 3) [4]. Right-sided colonic diverticulitis in many respects differs from its left-sided cousin. Diverticula of the right colon are usually congenital, solitary, true diverticula containing all bowel wall layers. The fecoliths within these diverticula are larger and the diverticular neck is wider. There is no hypertrophy of the muscularis of the right colonic wall . Puylaert JB in a study of 110 patients with right colonic diverticulitis clearly show, through US and CT, that it invariably has a favorable course and never leads to free perforation or large abscesses. Although relatively rare (left:right = 15:1), it is crucial to make a correct diagnosis since the clinical symptoms of acute right lower quadrant pain may lead to an unnecessary appendectomy or even right hemicolectomy.

## Conclusions

US and CT are both useful in diagnosis of diverticolitis, and their sensibility and specificity are similar. However, CT may be more likely to identify alternative causes of abdominal pain and it is essential for investigating complicated diverticular disease especially where there are diffuse signs and clinical suspicion of secondary peritonitis. [52] Instead in most uncomplicated cases the experienced sonographer may quickly confirm a diagnosis guided by the clinical signs. US is to be recommended in premenopausal women, and in young people to reduce dose exposure.

## Competing interests

The authors declare that they have no competing interests.

## Declarations

This article has been published as part of *Critical Ultrasound Journal* Volume 5 Supplement 1, 2013: Topics in emergency abdominal ultrasonography. The full contents of the supplement are available online at http://www.criticalultrasoundjournal.com/supplements/5/S1. Publication of this supplement has been funded by the University of Molise, University of Siena, University of Cagliari, University of Ferrara and University of Turin.
